# Retrospective Analysis of Fondaparinux and Low-Molecular-Weight Heparin in the Treatment of Women With Recurrent Spontaneous Abortion

**DOI:** 10.3389/fendo.2021.717630

**Published:** 2021-10-14

**Authors:** Long Zhao, Shuqin Bi, Jinhua Fu, Lijuan Qi, Lin Li, Yinghui Fu

**Affiliations:** ^1^ Department of Nephrology, The Affiliated Hospital of Qingdao University, Qingdao, China; ^2^ Department of Obstetrics, Qingdao Jinhua Gynecology Hospital, Qingdao, China

**Keywords:** thrombelastography, fondaparinux, low-molecular-weight heparin, anticoagulation, recurrent spontaneous abortion

## Abstract

**Background:**

To compare the clinical efficacy of fondaparinux and LMWH and provide clinical evidence for the effectiveness of fondaparinux in the treatment of recurrent spontaneous abortion caused by PTS.

**Methods:**

A retrospective analysis was conducted for 120 patients diagnosed with a recurrent spontaneous abortion caused by PTS in Qingdao Jinhua Women’s Hospital from March 2019 to April 2020. The patients were divided into two groups: 68 cases in the control group, treated with LMWH, 52 cases in the observational group, treated with fondaparinux. The pregnancy outcomes and adverse reactions between the two groups of recurrent miscarriage patients were compared.

**Results:**

No significant difference was detected in the general data between the two groups of patients before treatment (P>0.05). In the observational group, the R value was increased, and the α and MA values were decreased after three months of treatment compared to those before treatment (P*<*0.05). In the control group, the R value was increased, and the MA value was decreased after three months of treatment compared to those before treatment (P<0.05). After treatment, no significant difference was observed in the pregnancy outcome between the two groups (P>0.05). The total adverse reaction rate of the fondaparinux group was lower than that of the LMWH group (P<0.05).

**Conclusions:**

In this study, no significant difference was detected in the pregnancy outcome between fondaparinux and LMWH in the treatment of recurrent spontaneous abortion caused by PTS, but fondaparinux had a low occurrence rate of adverse reactions and high safety.

## Introduction

Recurrent spontaneous abortion (RSA) is referred to two or more consecutive pregnancy failures with the same partner (1). Studies have shown a vicious circle in patients with recurrent spontaneous abortion during re-pregnancy. The more abortions occurred, the higher the probability of abortion after re-pregnancy. Patients who had a history of more than four spontaneous abortions due to lack of proper treatment had a re-abortion rate of about 60% after pregnancy (2). Among the many factors that promote RSA, the prethrombotic state (PTS) (3) causes RSA caused, and anticoagulation is the first choice for treatment. PTS is a pathological hypercoagulable state, which refers to a pathological process of dysfunction or disturbance of coagulation, anticoagulation and fibrinolysis system caused by a variety of factors, and has a variety of hematological changes that can easily lead to thrombosis. Hereditary thrombosis refers to the genetic tendency of venous thrombosis. The most common causes are coagulation factor V; Leiden (FVL) mutations and prothrombin gene mutations (PGM), which account for 50% to 60% of Caucasian cases. Protein S deficiency, protein C deficiency and antithrombin (AT) deficiency accounted for the vast majority of other cases. Although first-time miscarriages are usually caused by chromosomal defects, about 55% of recurrent miscarriages are caused by procoagulant defects that induce thrombosis and infarction of placental vessels (4). During anticoagulation treatment, thrombelastography (TEG) reflects the coagulation process. Also, a comprehensive evaluation of the formation of blood clots, coagulation factors, platelet count, and fibrinolysis was conducted. Some studies have shown that anticoagulation therapy with low-molecular-weight heparin (LMWH) and aspirin improve pregnancy outcomes (5, 6). Due to the molecular mechanism of LMWH, adverse reactions, such as skin allergy and heparin-induced thrombocytopenia in pregnant women, are inevitable (7). When pregnant women at high risk of thrombosis develop heparin intolerance, the selection of anticoagulant therapy is limited. Fondaparinux is the first synthetic selective inhibitor of factor Xa. Reportedly, it has no bleeding tendency and thrombosis and could be used as an anticoagulant in pregnant women with heparin allergy (8–11). Thus, the present study evaluated the effects of fondaparinux and LMWH on the pregnancy outcomes and adverse reactions in the treatment of patients with a recurrent spontaneous abortion caused by PTS, thereby assessing the efficacy of fondaparinux on the RSA caused by PTS.

## Materials and Methods

### Patients

A retrospective analysis was conducted on 120 patients, including 68 cases in the control group and 52 cases in the observational group, diagnosed with recurrent spontaneous abortion at Qingdao Jinhua Women’s Hospital from March 2019 to April 2020. The diagnostic criteria were as follows: (1) The diagnostic criteria for RSA were proposed by the American Society of Reproductive Medicine, i.e., two or more consecutive pregnancy failures with the same partner (1). (2) Judgment of PTS revealed that there was currently no unified judgment standard in China. According to the “Expert Consensus on the Diagnosis and Treatment of Recurrent Spontaneous Abortion” published by the Chinese Journal of Obstetrics and Gynecology (12), the laboratory examination indicators related to the PTS were coagulation-related tests [thrombin time (TT), activated partial thromboplastin time (APTT), prothrombin time (PT), fibrinogen, and D-dimer], related autoantibodies [anticardiolipin antibody (ACA), anti-β2 glycoprotein 1 (β2GP1) antibody, lupus anticoagulant (LA)], and homocysteine (Hcy). In addition, protein C, protein S, factor XII, antithrombin III; (AT-III), and other PTS markers can also be detected. The inclusion criteria were as follows: I patients who had two or more consecutive pregnancy failures with the same partner; II patients whose laboratory examination met the criteria of PTS; III hysteroscopy or ultrasound examination of the uterus and double appendixes did not find any uterine abnormalities; ④ the chromosomal karyotypes of the couples were normal; ⑤ no contraindication to anticoagulant medication was observed. The exclusion criteria were as follows: ①abnormal reproductive endocrine hormones with pre-pregnant diabetes and thyroid disease; ② infectious diseases; ③ patients who had not received anticoagulation therapy; ④ drug allergy; ⑤ cases with incomplete data.

### Design of the Study

The clinical medication was based on the recommendations proposed by the “Expert Consensus on the Diagnosis and Treatment of Recurrent Spontaneous Abortion” (12). Based on the first trimester, blood β-hCG was used to diagnose pregnancy, and the drugs were begun to be administrated. Patients in the control group were subcutaneously injected with bemiheparin sodium (National Medicine Standard H20140019, Laboratorios Farmacéuticos ROVI, SA, 0.2 mL:3500 IU), once per day. Patients in the observational group were subcutaneously injected with fondaparinux sodium (National Medicine Standard H20183122, Jiangsu Hengrui Pharmaceutical Co., Ltd, 0.5 mL:2.5 mg) once a day. The drugs were stopped when monitoring of the two groups of patients showed that the fetal development was adequate, and the abnormal indicators related to the prethrombotic state returned to normal. After the drug administration was stopped, indicators related to the prethrombotic state were re-examined regularly, and the growth and development of the fetus were monitored simultaneously. Patients with abnormalities were evaluated by the doctor, and the drug administration was restarted, and the treatment continued throughout the pregnancy. The medication was stopped 24 h before the termination of pregnancy. During the use of fondaparinux and LMWH, adverse drug reactions were monitored.

Except for fondaparinux and LMWH, the other anticoagulant drugs were not used, and no difference was detected in the use of antiplatelet drugs, such as aspirin 75 mg/d. RSA patients with positive antinuclear antibodies were treated with prednisone 5 mg/d, combined with cyclosporine 100 mg/d and hydroxychloroquine sulfate 300 mg/d.

### Observational Indicators

1. The pregnancy outcomes, including stillbirth, abortion, premature delivery, and full-term delivery were compared between the two groups, and the live birth rate of the patients in the two groups was evaluated as follows: Live birth rate = [(premature delivery + term delivery)/number of patients] ×100%.
2. The difference in the parameters of thromboelastography (TEG) after three months of treatment in the two groups was compared to those before the treatment.
3. The occurrence of adverse reactions between the two groups were compared. The adverse reactions included vaginal bleeding, thrombocytopenia, ecchymosis, and gastrointestinal reactions.

### Statistical Methods

The data in this study were analyzed and processed using SPSS20.0 statistical software. Measurement data were expressed as (mean ± standard deviation), and t-test was used for comparison. Count data were expressed as rate (%), and X^2^ test was used for comparison. The test level was α=0.05, and P<0.05 indicated statistical significance.

## Results

### Comparison of General Data Between the Two Groups of Patients

The comparison of general data between the two groups of patients showed no statistically significant difference in age, the number of spontaneous abortions, pregnancy times, time of drug use, and TEG parameters before the treatment, indicating that the two groups were similar (P>0.05; **Tables 1** and **2**).

**Table 1:** Comparison of general data between the two groups of patients (mean ±standard deviation).

Groups	Age (years)	Spontaneous abortion (times)	Pregnancy times (times)	Time of drug use (days)
Observational group (n=52)	31.33 ± 4.22	2.75 ± 0.81	3.19 ± 0.97	167.54 ± 11.67
Control group (n=68)	31.60 ± 4.08	2.69 ± 0.90	3.01 ± 1.01	168.09 ± 12.23
t-value	0.361	0.369	0.968	0.249
P-value	0.718	0.713	0.335	0.804

**Table 2:** Comparison of parameters of pre-treatment thromboelastography between the two groups of patients (mean ± standard deviation).

Before treatment	R value	K value	a value	MA value
Observational group	5.69 ± 1.04	2.13 ± 0.47	64.23 ± 5.30	62.74 ± 4.68
Control group	5.59 ± 1.02	2.03 ± 0.48	64.29 ± 4.32	63.18 ± 3.82
t-value	0.348	0.397	0.095	1.006
P-value	0.730	0.699	0.925	0.315

### Comparison of the Parameters of TEG in the Observational Group Three Months After Treatment and Before Treatment

For patients in the observational group, the R value was increased and the α and MA values were decreased significantly after three months of fondaparinux sodium treatment compared to those before treatment (P<0.05), while no significant difference was detected in the K value (P>0.05; **Table 3**).

**Table 3:** Comparison of TEG parameters three months after treatment and before treatment in the observational group (mean ± standard deviation).

Time points	R value	K value	a value	MA value
Before treatment	5.69 ± 1.04	2.13 ± 0.47	64.23 ± 5.30	62.74 ± 4.68
Three months after treatment	6.03 ± 1.06	2.01 ± 0.56	63.49 ± 5.41	61.85 ± 4.72
t value	3.898	1.588	5.592	7.627
P-value	0.000	0.114	0.000	0.000

### Comparison of TEG Parameters in the LMWH Group Three Months After Treatment and Before Treatment

For patients in the control group, the R value was increased, and the MA value was decreased significantly after three months of LMWH treatment compared to those before treatment (P<0.05), while no significant differences were detected in the K and α values after three months of LMWH treatment compared to those before treatment (P>0.05; **Table 4**).

**Table 4:** Comparison of TEG parameters three months after treatment and before treatment in the LMWH group (mean ± standard deviation).

Time points	R value	K value	a value	MA value
Before treatment	5.59 ± 1.02	2.03 ± 0.48	64.29 ± 4.32	63.18 ± 3.82
Three months after treatment	5.80 ± 0.79	1.94 ± 0.55	64.11 ± 4.14	62.32 ± 4.45
t value	2.900	1.274	1.699	8.512
P-value	0.004	0.204	0.089	0.000

### Comparison of Pregnancy Outcomes Between the Two Groups

After treatment, no stillbirth cases were observed in the two groups. The observational and control groups had 2 and 6 cases of abortion, respectively; 2 and 9 cases of premature births were detected in the two groups, respectively; 48 and 53 cases of full-term births in the observational group and control group, respectively. The live birth rate in the observational group was 96.15%, which was slightly higher than 91.18% in the control group, albeit not significantly with respect to the pregnancy outcome between the two groups (c^2 =^ 1.173, P=0.463; **Table 5**).

**Table 5:** Comparison of pregnancy outcomes between the two groups (n, %).

Groups	Stillbirth	Abortion	Premature delivery	Term birth	Live birth rate
Observational group (n=52)	0	2 (3.85)	2 (3.85)	48 (92.30)	50 (96.15)
Control group (n=68)	0	6 (8.82)	9 (13.24)	53 (77.94)	62 (91.18)
c^2^ value					1.1 (73)
P-value					0.4 (63)

### Occurrence of Adverse Reactions in the Two Groups

The data showed that three and two cases of vaginal bleeding in the control group and observational group, respectively. The amount of bleeding was less, with slight secretion. The symptoms disappeared after bed rest. In addition, one case of thrombocytopenia was noted in the control group and none in the observational group. During the treatment period, six cases of ecchymosis occurred after subcutaneous injection of LMWH sodium in the control group, and no ecchymosis occurred in the observational group after subcutaneous injection of fondaparinux sodium. Next, we observed one and three gastrointestinal reaction cases in the observational and control groups, respectively. The total adverse reaction rate in the observational and control groups was 5.76% and 19.12%, respectively. Comparison between the two groups showed that the total adverse reaction rate after fondaparinux sodium treatment was significantly lower than that of LMWH, and the difference was statistically significant (c^2 =^ 6.892, P=0.042; **Table 6**).

**Table 6:** Occurrence of adverse reactions in the two groups (n, %).

Groups	Vagina bleeding	Thrombocytopenia	Ecchymosis	Gastrointestinal reaction	Total occurrence rate
Observational group (n=52)	2 (3.84)	0 (0)	0 (0)	1 (1.92)	3 (5.76)
Control group (n=68)	3 (4.41)	1 (1.47)	6 (8.82)	3 (4.41)	13 (19.12)
c^2^ value					6.892
P-value					0.042

## Discussion

In recent years, the number of patients with recurrent spontaneous abortion has increased gradually, and women with multiple abortion history were under tremendous physical and mental stress. Therefore, effective treatment regimens for preventing abortion at this stage are under intensive focus research. The occurrence of recurrent abortion was related to the abnormal blood coagulation function in the patient’s body, and PTS was also a key factor causing recurrent abortion in patients (13, 14). The mechanism of abortion caused by PTS was such that the hypercoagulable state of blood during pregnancy altered the blood flow state of the uterine placenta and microthrombosis of the uterine spiral artery or villi vessels. These factors resulted in poor uterine fetal blood circulation, infarction, and embryonic ischemia and hypoxia, leading to poor development or abortion (15). TEG reflects the dynamic changes of coagulation and fibrinolysis of the whole blood and is the only method that reflects the interaction between coagulation factors, platelets, and fibrin, and other cellular components.

Anticoagulation therapy is currently recognized as the most effective treatment method for patients with PTS. The commonly used anticoagulation drugs mainly include LMWH, fondaparinux sodium, and warfarin (15). Warfarin can pass through the placenta, adversely affect the fetus, and even cause fetal malformations, leading to an increased probability of fetal death in the uterus, and hence should be used with caution (2, 3). LMWH was conducive to the strengthening and improved differentiation and invasion speed of trophoblasts (16). However, this event triggered a series of adverse reactions through type I hypersensitivity, vasculitis-induced skin necrosis (type III reaction), or heparin-induced thrombocytopenia (HIT) (15–17). Fondaparinux is a synthetic pentasaccharide bound to antithrombin and inhibitor Xa but does not inhibit thrombin (18). Compared to heparin, fondaparinux did not increase the production of human monocytes and whole blood cytokines induced by lipopolysaccharide and did not cause local immune reactions, leading to erythema and skin damage. A large number of studies have shown that fondaparinux has the same curative effect as LMWH in preventing venous thromboembolism (VTE) and treating deep vein thrombosis (DVT). Moreover, pulmonary embolism (PE) in a major orthopedic surgery but does not increase major bleeding (19, 20). The American College of Chest Physicians recommended that at level 2C, pregnant women who had a severe allergic reaction to heparin (such as HIT) and could not receive dalteparin sodium should use fondaparinux sodium for treatment (21).

For the anticoagulation study of fondaparinux in the field of Gynecology, Wijesiriwardana et al. (11)confirmed that patients received a daily subcutaneous injection of fondaparinux 2.5 mg due to acute severe skin reactions to LMWH. Mazzolai et al. (10) reported a case of known protein S deficiency, with a family history of DVT and thromboembolic disease (TED). Anticoagulant prophylaxis was required during pregnancy. The patient showed severe skin allergies to LMWH and unfractionated heparin (UFH). She received fondaparinux treatment until delivery. Neither allergic reaction nor thromboembolic events or abnormal bleeding were observed at the injection site. In a study by Winger et al. (22), the efficacy of fondaparinux and enoxaparin on patients with recurrent spontaneous abortion was compared. It was found that the pregnancy success rate of patients receiving fondaparinux sodium and enoxaparin treatment was 59% and 58%, respectively. In another randomized controlled trial there was a reduction in risk of adverse pregnancy outcome when administering low-molecular-weight heparin versus placebo among women with a thrombophilia and a history of delivery before 34 weeks of gestation with hypertensive disease, or small-for-gestational-age infants, or both (risk difference, 8.7%; 95% CI, 1.9–15.5%) (23).RSA with APS, PTS and autoimmune diseases is the first choice for LMWH anticoagulation therapy (or combined with aspirin) (24). Most studies have reported that anticoagulant therapy can improve the pregnancy outcome of patients with URSA, RBP and RPF (25, 26)Warfarin and hirudin cross the placenta and are associated with embryo and fetal toxicities. Lagrange et al. (27) did not observe placental transfer of fondaparinux in an *in vitro* model with the use of dually perfused human cotyledon. On the other hand, fondaparinux has been reported to pass *in vivo* the placental barrier, resulting in low but measurable anti-factor Xa activity in umbilical-cord blood,so that a potential hazard cannot be ruled out (28, 29). No statistically significant difference was observed in newborn weight and gestational age at delivery, and no congenital disabilities, severe bleeding-related complications, or severe allergic reactions were observed. However, these studies were conducted in European and American populations before 2010. At present, there is no large-scale clinical study of fondaparinux in the field of gynecological anticoagulation in China. In this study, a retrospective analysis of the different anticoagulant medication regimens for women with a history of RSA revealed that the TEG parameters before treatment in women with a history of RSA were abnormal, which in turn led to a high risk of abortion when they were pregnant again. Both heparin and LMWH were effective in improving the hypercoagulable state of patients after three months of treatment. Compared to before treatment, the R value was increased, and the MA value was decreased. In addition, fondaparinux significantly reduced the α value of patients. Regarding pregnancy outcome, the live birth rate of the fondaparinux group was 96.15%, which was slightly higher than the 91.18% in the LMWH group, suggesting that both fondaparinux sodium and LMWH sodium improves the pregnancy outcome of patients with recurrent spontaneous abortion. Furthermore, the total adverse reaction rate of fondaparinux was 5.76% and 19.12% in the LMWH group. Thus, the total adverse reaction rate of fondaparinux was lower than that of the LMWH group, confirming that it is highly safe in the treatment of relapsed patients. This phenomenon may be related to the fact that fondaparinux does not inhibit thrombin and does not induce local immune reactions that cause erythema and skin damage but not thrombocytopenia.

## Conclusions

In conclusion, both fondaparinux and LMWH treatment improve the hypercoagulable state of the patient in the treatment of PTS-induced RSA. Although the pregnancy outcome was equivalent, the occurrence rate of adverse reactions was lower in the fondaparinux group than the heparin group, reflecting that the treatment was safe and applicable. No difference was observed in the pregnancy outcome in our analysis, There by necessitating large clinical trials to confirm these findings.

## Data Availability Statement

The original contributions presented in the study are included in the article/supplementary material. Further inquiries can be directed to the corresponding author.

## Ethics Statement

The studies involving human participants were reviewed and approved by China Ethics Committee of Registering Clinical Trials. Written informed consent for participation was not required for this study in accordance with the national legislation and the institutional requirements.

## Author Contributions

Conception and design, LZ, SB, LQ, and JF. Administrative support, LZ. Provision of study materials or patients, LQ, SB, and YF. Collection and assembly of data, LQ, SB, and YF. Data analysis and interpretation, LZ, SB, LQ, LL, and JF. All authors contributed to the article and approved the submitted version.

## Funding

National Natural Science Foundation of China (81800601), China Postdoctoral Science Foundation (2019M652333), China health promotion foundation (XM_2020_011_0245_09), Qingdao Key Health Discipline Development Fund.

## Conflict of Interest

The authors declare that the research was conducted in the absence of any commercial or financial relationships that could be construed as a potential conflict of interest.

## Publisher’s Note

All claims expressed in this article are solely those of the authors and do not necessarily represent those of their affiliated organizations, or those of the publisher, the editors and the reviewers. Any product that may be evaluated in this article, or claim that may be made by its manufacturer, is not guaranteed or endorsed by the publisher.
